# Factors influencing the survival of outmigrating juvenile salmonids through multiple dam passages: an individual‐based approach

**DOI:** 10.1002/ece3.2326

**Published:** 2016-07-25

**Authors:** Timothy Elder, Christa M. Woodley, Mark A. Weiland, Angela L. Strecker

**Affiliations:** ^1^ Department of Environmental Science and ManagementPortland State UniversityPO Box 751PortlandOregon97207; ^2^ Environmental LaboratoryUS Army Engineer Research and Development Center3909 Halls Ferry RdVicksburgMississippi39180; ^3^ Pacific Northwest National Laboratory390 Evergreen Dr.PO Box 241North BonnevilleWashington98639

**Keywords:** Acoustic transmitters, Chinook salmon, dissolved gas, involuntary spill, random forest, steelhead trout

## Abstract

Substantial declines of Pacific salmon populations have occurred over the past several decades related to large‐scale anthropogenic and climatic changes in freshwater and marine environments. In the Columbia River Basin, migrating juvenile salmonids may pass as many as eight large‐scale hydropower projects before reaching the ocean; however, the cumulative effects of multiple dam passages are largely unknown. Using acoustic transmitters and an extensive system of hydrophone arrays in the Lower Columbia River, we calculated the survival of yearling Chinook salmon (*Oncorhynchus tshawytscha*) and steelhead (*O. mykiss*) passing one, two, or three dams. We applied a unique index of biological characteristics and environmental exposures, experienced by each fish individually as it migrated downstream, in order to examine which factors most influence salmonid survival. High outflow volumes led to involuntary spill in 2011 and created an environment of supersaturated dissolved gas concentrations. In this environment, migrating smolt survival was strongly influenced by barometric pressure, fish velocity, and water temperature. The effect of these variables on survival was compounded by multiple dam passages compared to fish passing a single dam. Despite spatial isolation between dams in the Lower Columbia River hydrosystem, migrating smolt appear to experience cumulative effects akin to a press disturbance. In general, Chinook salmon and steelhead respond similarly in terms of survival rates and responses to altered environmental conditions. Management actions that limit dissolved gas concentrations in years of high flow will benefit migrating salmonids at this life stage.

## Introduction

Anthropogenic alterations within and adjacent to freshwater ecosystems have caused habitat degradation and loss, resulting in the decline of many aquatic species (Poff et al. 1997; Dudgeon et al. 2006). Pacific salmon populations face serious human‐mediated threats across multiple life stages and throughout much of their distribution (Bigler et al. 1996; Budy et al. 2002). The Columbia River Basin in western North America is one of the most dammed river systems globally and has experienced extensive anthropogenic alterations that have affected many organisms, including the early life stages of salmonids (Gresh et al. 2000; Smith et al. 2003).

The creation of the Federal Columbia River Power System (hydroelectric dams and reservoirs) has significantly altered the physical, chemical, and biological structure of the Columbia River, including increases in water temperature, total dissolved gas, and predation pressure; altered flow regimes; and disrupted salmonid migration (Raymond 1979; Giorgi et al. 1997; Bickford and Skalski 2000; Petersen 2001; Smith et al. 2003; Kuehne and Olden 2012). Repeated delays in smolt outmigration, injury, stress, and disorientation caused during passage through hydroelectric power facilities, and their associated reservoirs can manifest directly as mortality or indirectly due to increased susceptibility to predation or disease (Abernethy et al. 2001; Čada 2001). Of particular importance are the cumulative effects that occur in situ as juvenile salmon migrate through multiple hydroelectric power projects (Schaller et al. 1999).

While Pacific salmon have evolved a suite of life‐history traits that provide resilience through unpredictable environmental variability, there is much to learn about how disturbances caused by hydroelectric dams within altered systems affect the ecology and survival of migrating smolts (Hicks et al. 1991). We are particularly interested in whether conditions created by the hydrosystem in the Lower Columbia River constitute a pulse disturbance (i.e., acute stress) or a press disturbance (i.e., chronic stress) for migrating smolts (Reeves et al. 1995). If hydroelectric dams create a pulse disturbance within the migration corridor, acute exposures to areas of deleterious environmental conditions should not result in cumulative effects for fish passing multiple dams. On the other hand, if conditions within the hydrosystem create a press disturbance, we would expect chronic exposures and cumulative negative effects for smolt passing multiple dams.

We identified biological and environmental variables that influenced yearling Chinook salmon (*Oncorhynchus tshawytscha*) and steelhead (*O. mykiss*) passing one, two, and three dams in the Lower Columbia River and determined each variables' importance in terms of survival. Previous research has investigated the effect of environmental variables on groups of tagged fish, often passing a single dam and reservoir, but our study is the first to our knowledge to compare how the survival of individual fish, implanted with acoustic transmitters, is influenced by environmental factors for fish passing one, two, or three dams. We are seeking a better understanding of how the importance of variables changes for fish passing different numbers of dams and associated reservoirs during outmigration in order to better appreciate the dynamic and altered ecological processes influencing salmonids in the Columbia River Basin and other impounded systems.

We hypothesize that (1) the survival of fish passing one, two, and three dams will be influenced by different biological and environmental variables (Schaller and Petrosky 2007); (2) variables affecting fish survival through multiple dams will show cumulative effects (press disturbance) compared to fish passing a single dam (pulse disturbance) (Petrosky and Schaller 2010); and (3) based on the similar life cycle stages, juvenile Chinook salmon and steelhead will respond similarly to altered environmental variables (Haeseker et al. 2012).

## Methods

### Study area and experimental design

The Columbia River Basin occupies an area of 660,480 km^2^ and is the second largest river system, by volume, in the United States. Our study area covered the mainstem Lower Columbia River from river kilometer (rkm) 161–390, including three hydroelectric power projects: Bonneville Dam (BON, rkm 234), The Dalles Dam (TDA, rkm 309), and John Day Dam (JDA, rkm 347) (see Appendix S1 in Supporting Information). Each dam has multiple fish passage routes that allow migrating smolt downstream. All dams are equipped with a powerhouse, a spillway, and either a juvenile bypass structure (BON, JDA), sluiceway (BON, TDA) or surface weir (JDA) (Ploskey et al. 2012).

Yearling spring‐run Chinook salmon and steelhead outmigrating between April 25, 2011, and May 28, 2011, were collected at the John Day Dam smolt monitoring facility. Chinook salmon (*n *=* *5208) and steelhead (*n *=* *5175) smolt included in the study ranged in size from 95 to 299 mm (fork length), were not moribund (i.e., not expected to die within 24 h), had no deformities or injuries that would prevent tag insertion and surgical closure (i.e., pronounced spinal deformation, large wounds on abdomen), and had not been previously tagged (i.e., PIT, acoustic or radio transmitter). Fish were surgically implanted with (1) a passive integrated transponder (PIT) (HPT12, BioMark, Boise, ID) and (2) a Juvenile Salmon Acoustic Telemetry Systems (JSATS) acoustic microtransmitter (Model SS130; Advanced Telemetry Systems, Isanti, MN). The JSATS transmitters actively broadcast a unique 156‐db acoustic signal with a pulse interval of three seconds, but have a limited battery powered life span (Appendix S2). All fish were released into the mainstem Lower Columbia River at one of five release points after an 18‐ to 24‐h recovery period (see Skalski et al. 2012a,b,c for details regarding permitting and fish handling). Fish handling, surgical procedures, and transportation experience were standardized between all fish release groups and sites.

As juveniles migrated downstream from release points, they were detected in‐river passing up to six autonomous hydrophone arrays (Appendix S1). Each detection array consisted of three to nine hydrophones capable of detecting and recording the unique acoustic signal transmitted from each implanted fish. Additionally, each hydroelectric power project was retrofitted with 83–98 hydrophones on the forebay of each dam. Fish detections were considered confirmed if a unique acoustic signal was detected four times within a 48‐sec period at a single detection array. Fish that were detected at an array and then not detected at any subsequent arrays were considered mortalities of unknown cause (i.e., dam passage‐related mortality, predation, dropped tags, or termination of migration). To ensure that all smolts migrating through the hydroelectric power system had adequate time to pass, the in‐river and dam‐mounted detection arrays were monitored through June 2011.

Our research utilized a subset of data collected in 2011 by Pacific Northwest National Laboratories (PNNL) as part of the 2008 Federal Columbia River Power System Biological Opinion Compliance Monitoring. Details specific to the experimental designs, methodologies, and statistical assumptions for each hydroelectric power project can be found in Johnson et al. (2012), Ploskey et al. (2012), Weiland et al. (2013), and Skalski et al. (2012a,b,c). As a result of different subsampling, analysis, and modeling techniques, survival estimates differ from previously published compliance reports.

### Environmental data

The Columbia Basin Research Data Acquisition in Real Time (DART) program collects hourly environmental data on total project outflow discharge (m^3^·sec^−1^), spillway discharge (m^3^·sec^−1^), water temperature (°C), total dissolved gas (%), and atmospheric barometric pressure (mmHg) at the forebay of each dam in the Lower Columbia River hydrosystem (http://www.cbr.washington.edu/dart). Ranges of environmental variables at each dam are reported in Appendix S3. For each individual fish, we created an averaged index representing the unique environmental conditions experienced by that fish based on the time between its release and last detection through a given array. This index of averaged hourly environmental variables was applied to each fish based on which dams were passed (e.g., fish passing BON were assigned the averaged hourly environmental variables from BON, while fish passing JDA, TDA, and BON were assigned the averaged environmental variables from all dams over the time period between release and last detection).

### Data analysis

We used random sampling to assign fish into treatments that passed through different numbers of dams (i.e., passing one, two, or three dams), which were stratified by release date. Random sampling was conducted without replacement (i.e., all fish within each treatment group are independent of fish in other treatment groups), allowing for comparison between dam passages. The one dam treatment group was comprised of fish passing only Bonneville, The Dalles, or John Day Dams (Table 1). The two dam treatment group included fish passing John Day and The Dalles Dam and fish passing The Dalles Dam and Bonneville. The three dam treatment group passed all dams in the system (Table 1). This experimental design controlled for differences in survival caused by environmental and structural differences between each dam and changes in environmental conditions throughout the season.

**Table 1:** Description of dam passage experience for fish, including which dams were passed, release sites and detection arrays, the source of environmental data that was applied to each fish, the number of smolt, and survival estimates. Smolt passing one and two dams were combined for analysis from fish passing different dams (e.g., one dam passage included fish passing BON, TDA, and JDA and two dam fish passed JDA–TDA and TDA–BON). Pearson's chi‐squared tests were performed within treatment groups to test whether fish passing a single dam (either BON, TDA, or JDA) or two dams (JDA–TDA and TDA–BON) had statistically different survival estimates (shared italic letters indicate no significance, while different letters indicate significant differences within each dam passage experience). Analysis of variance was performed between combined passages (i.e., one, two, or three dams) and controlled for release date. Post hoc Tukey's honest significant difference test was used to identify differences between survival based on passage experience (bold letters)

	Dams passed	Release and detection arrays (rkm)	Source of environmental data	Chinook (*n*)	Chinook survival (%)	Steelhead (*n*)	Steelhead survival (%)
1 Dam Passage	BON	275–161	BON	800	83.3^*b*^	794	92.8^*b*^
	TDA	325–275	TDA	976	85.8^*b*^	955	86.0^*a*^
	JDA	390–325	JDA	728	93.3^*a*^	737	94.6^*b*^
Total				**2504**	**87.8** ^**a**^	**2486**	**90.7** ^**a**^
2 Dam Passage	TDA + BON	325–161	Average of TDA + BON	978	85.0^*a*^	985	88.1^*b*^
	JDA + TDA	390–275	Average of JDA + TDA	724	84.0^*a*^	734	83.9^*a*^
Total				**1702**	**83.8** ^**b**^	**1719**	**86.1** ^**ab**^
3 Dam Passage	JDA + TDA + BON	390–161	Average of JDA + TDA + BON	1002		970	
Total				**1002**	**81.7** ^**b**^	**970**	**84.3** ^**b**^

In order to confirm a basic assumption that fish passing different numbers of dams have different survival rates, a block design analysis of variance (ANOVA) was used to test the effect of number of dam passages (treatment; *n* = 3) on survival estimates for each species, while controlling for seasonal changes associated with release date (blocks; *n* = 16). A post hoc Tukey's honest significant difference (HSD) test was used to identify specific differences between treatment groups. Pearson's chi‐squared tests were used to determine whether significant differences exist between the survival of yearling Chinook salmon and steelhead.

For each fish, the predictor variables included the aforementioned physical variables (water temperature, outflow discharge, spillway discharge, total dissolved gas, and atmospheric barometric pressure), as well as fish migration rate (km·h^−1^: distance each fish travelled between release site and a specific detection array, divided by the time between release and detection at that array; Table 1), fish length (mm), and release date (day of year). In order to understand the relationships between predictor variables, a principal components analysis (PCA) was run on centered and scaled environmental data for Chinook passing one dam (Appendix S3). This subset of data had the largest number of observations and contained environmental data from all three dams in the Lower Columbia River hydrosystem, and thus, we believe was the most representative set of environmental conditions experienced by migrating smolt. The PCA indicated that the first four principal components explained 95.22% of the variance among the predictor variables (Appendix S3). The first principal component (PC1) explained 65.02% of the variance and was driven by positive correlations between release date, outflow discharge, spillway discharge, water temperature, and dissolved gas. The second and third principal components explained 12.97% and 11.95% of the variance, respectively, and were both driven by fish length and barometric pressure. Principal component 4 explained 5.25% of the variance and was dominated by fish velocity. Fish length, barometric pressure, and fish velocity were orthogonal in the PCA (i.e., uncorrelated) and were therefore interpreted with more confidence in subsequent analyses.

Random forest analyses were performed using the *randomForest* package in R (Liaw and Wiener 2014). Random forests utilize an ensemble bootstrapping technique, which produces a forest of classification trees, created and validated with randomly selected subsets of data. After producing 5000 trees for each species and each dam passage experience, variable importance was assessed based on the classification accuracy rates of all trees in that model (Cutler et al. 2007; Olden et al. 2008). The classification accuracy rate is the percent of fish that were correctly classified (PCC), where models with correct classifications >50% are considered better than random (Cutler et al. 2007). Cohen's Kappa statistic was calculated for each fish species and each dam passage experience to compare predicted and expected model accuracy, while accounting for model agreements due to random chance (Cutler et al. 2007). Kappa values range from −1 to 1, where values between 0.41 and 0.60 indicate moderate agreement, 0.61 and 0.80 indicate substantial agreement, and 0.81 and 1.0 indicate almost perfect agreement (Viera and Garrett 2005). We chose random forest analysis, instead of more familiar logistic regression, in order to retain biologically significant predictor variables. In past studies, biologically important variables have been excluded from traditional regression analysis based on multicollinearity with other biologically significant variables (Giorgi et al. 1997; Smith et al. 2003, Petrosky and Schaller 2010) (Appendix S3).

Random forest used two randomly selected predictor variables as candidates for each split during tree creation, thus substantially reducing the impact of correlated variables on the forest of 5000 trees. Variable importance was calculated using the Gini Index, a measure of node impurity calculated from the random forest, where large decreases in Gini Index indicate higher variable importance (De'ath and Fabricius 2000). Cross‐validated partial dependence plots were generated with the *interpretR* package in R (Ballings and Van den Poel 2016) and were used to evaluate the effect of each variable on survival while averaging out the effects of the other variables. For each dam passage experience, confidence intervals were created from 10 cross‐validated random forest models and represent the interquartile range. All analyses were conducted using R version 3.0.1 (R Core Team 2013).

## Results

There was a significant difference between the survival of yearling Chinook salmon passing one, two, and three dams after controlling for release date (block design ANOVA: *F*_2,16_ = 14.37, *P *<* *0.01). A post hoc Tukey's HSD test showed a significant difference between Chinook passing one and two, and one and three dams (*P *<* *0.05), but not between fish passing two and three dams (*P *=* *0.32). There was also a significant difference between the survival of steelhead passing different numbers of dams while controlling for release date (*F*_2,16_ = 4.46, *P *=* *0.02), but this difference was only significant for steelhead passing one and three dams (Tukey's HSD: *P *<* *0.05) (Appendix S2).

The survival of yearling Chinook salmon passing one, two, and three dams was 87.8%, 83.8% and 81.7%, respectively, while the survival of steelhead smolt passing one, two, and three dams was 90.7%, 86.1%, and 84.3%, respectively. There was not a significant difference between the survival of juvenile Chinook salmon and steelhead passing any number of dams (Pearson's chi‐squared test*, χ* ^2^ *= 1.83, P *=* *0.39), despite differences in fish length between these species (Chinook salmon mean = 148.3 mm (± 20.8SD), steelhead mean 203.7 mm (± 24.5SD); Welch two sample *t*‐test, *t *=* *−151.84, *P *<* *0.01).

**Figure 1:**
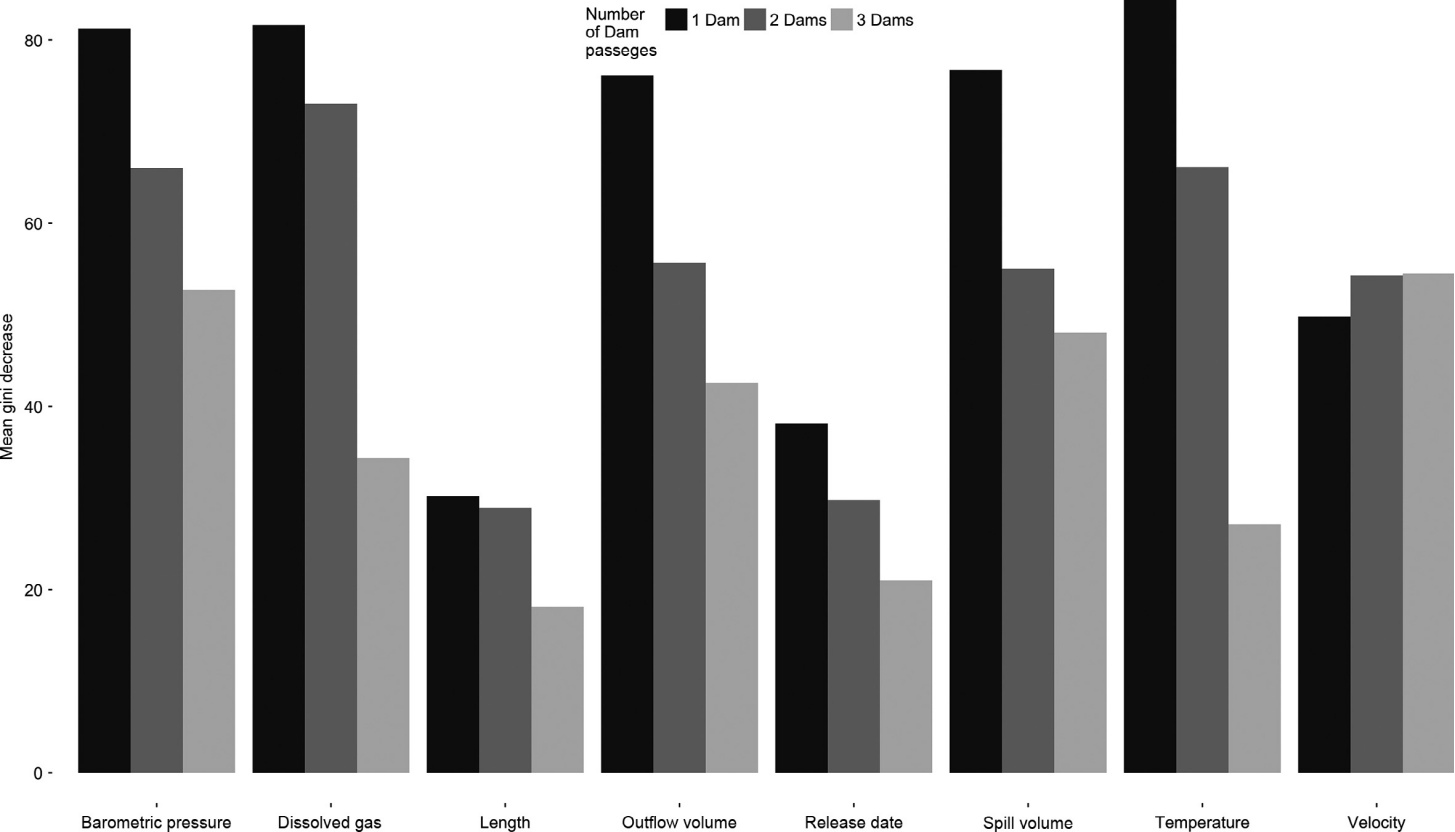
Variable importance for the survival of Chinook salmon passing through the Lower Columbia River hydroelectric power system for each individual random forest model (i.e., one, two, or three dams). Larger Gini values represent the most important variables regarding the survival of migrating smolts.

**Figure 2:**
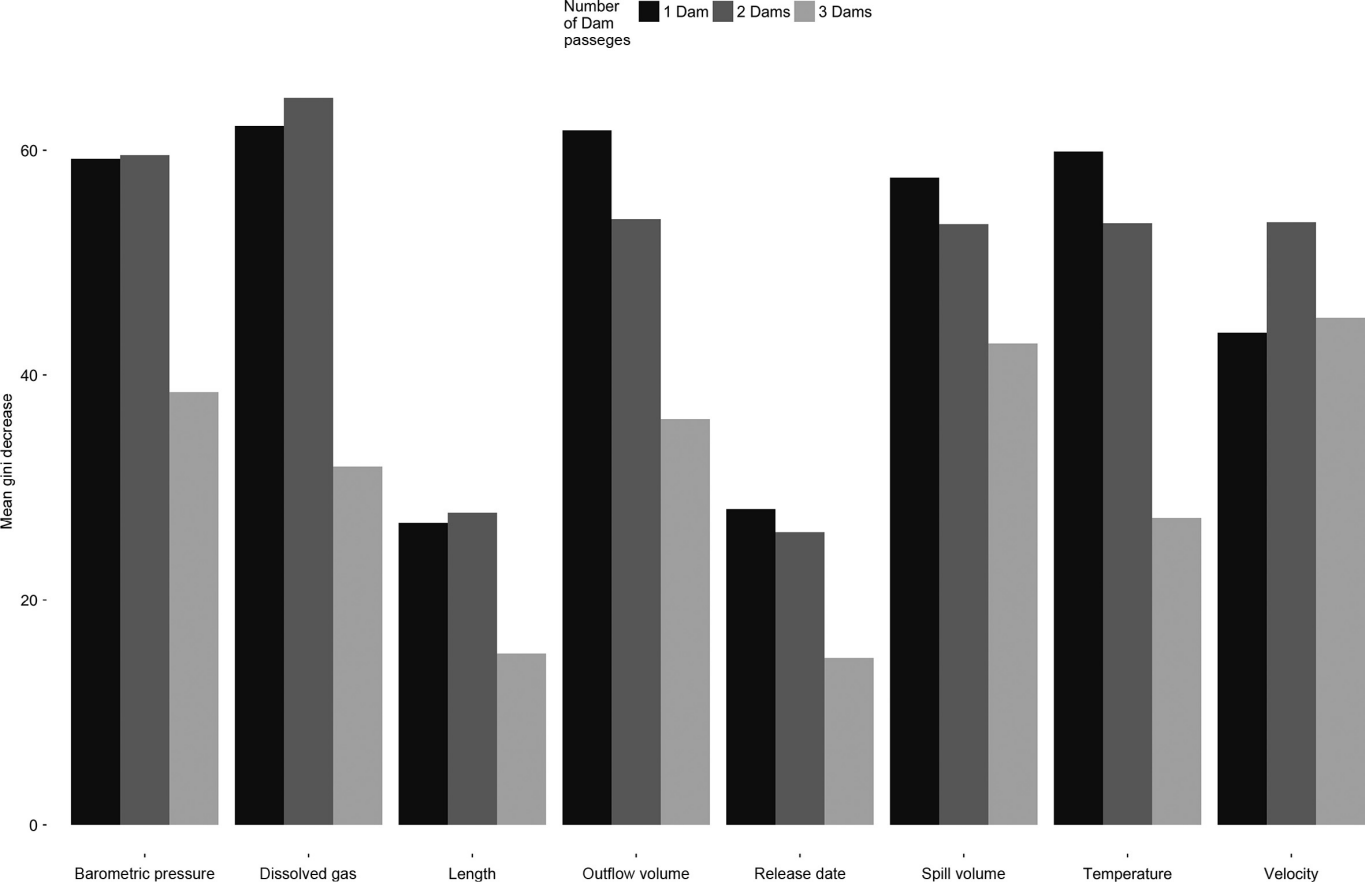
Variable importance for the survival of steelhead passing through the Lower Columbia River hydroelectric power system. Axis values as in Figure 1.

The survival of both species passing one or two dams was most influenced by water temperature, dissolved gas, outflow discharge, and barometric pressure in random forest models, whereas fish passing three dams were most influenced by spillway discharge, fish velocity, and barometric pressure (Figs 1 and 2). Random forest models for Chinook salmon and steelhead resulted in Cohen's Kappa statistics that ranged between 0.52 and 0.69, indicating moderate to substantial agreement between expected and predicted model accuracies (Table 2). For all Chinook and steelhead models, sensitivity (the models' ability to accurately predict survival) was greater than 98% and specificity (ability to predict mortality) ranged between 43% and 60% for Chinook and 39% and 50% for steelhead (Table 2).

**Table 2:** Performance of random forest models for yearling Chinook salmon and steelhead passing 1, 2, and 3 dams. Percent correctly classified (PCC) is the overall number of correctly classified model observations. Sensitivity is the percentage of times survival was correctly classified. Specificity is the percentage of times mortality was correctly classified. Cohen's Kappa statistic compares predicted model accuracy and expected model accuracy while accounting for agreement between models due to random chance

	Chinook salmon			Steelhead		
	1 Dam	2 Dams	3 Dams	1 Dam	2 Dams	3 Dams
PCC (%)	94.4	89.8	91.8	94.3	91.8	91.8
Sensitivity (%)	99.2	98.8	98.8	99.9	98.6	99.5
Specificity (%)	60.0	43.4	60.7	39.1	42.5	50.0
Cohen's Kappa	0.69	0.53	0.68	0.53	0.52	0.61

**Figure 3:**
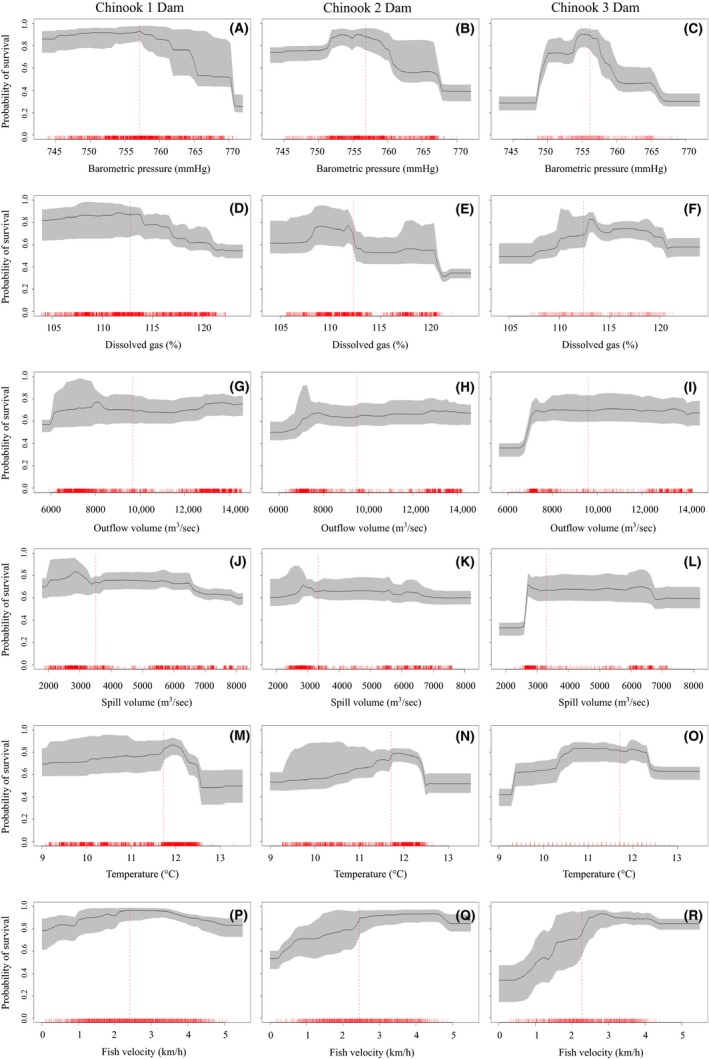
Partial dependence plots for Chinook salmon by variable and dam passage experience, generated from 10 cross‐validated random forest models. Partial dependence plots show the probability of survival for a given predictor variable, while averaging out the effects of the other predictor variables. Confidence intervals represent the interquartile range (gray), and vertical dashed line represents the median for each variable (red). Rug marks along the *x*‐axis indicate the number of fish experiencing those conditions. Plot areas at the extreme ends of the *x*‐axis, with few observations, should be interpreted cautiously.

**Figure 4:**
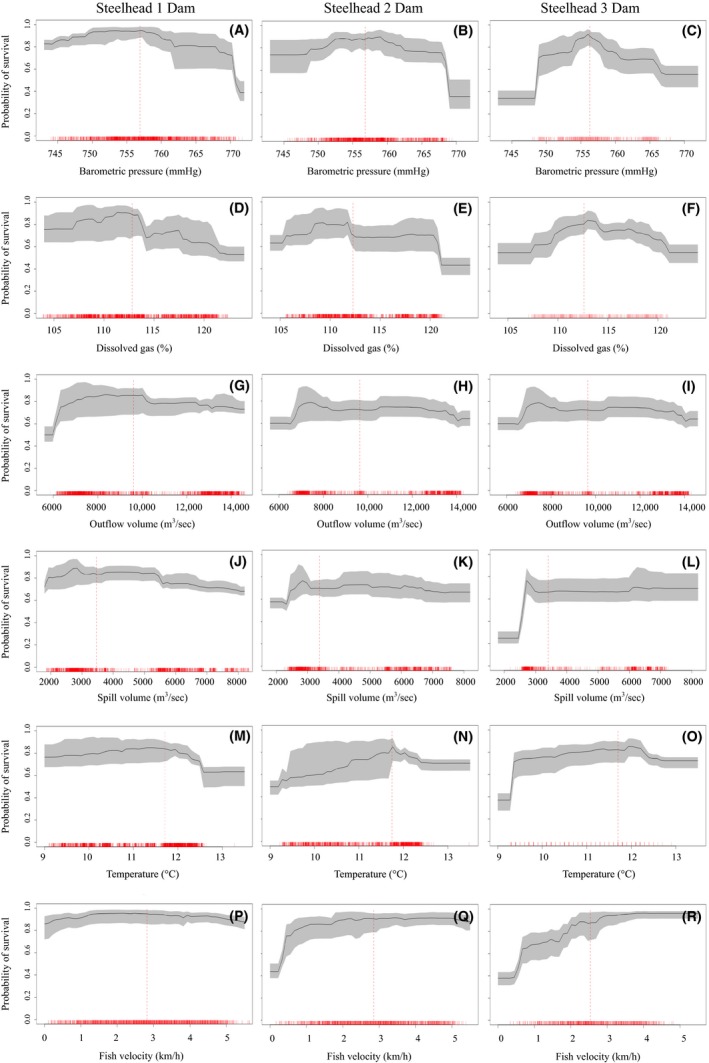
Partial dependence plots for steelhead by variable and dam passage. Symbols and axis values as in Figure 3.

Partial dependence plots generated from ten, cross‐validated random forest models show the partial effect of each predictor variable on the probability of survival while averaging out the effects of other variables. For both Chinook and steelhead passing one dam, barometric pressure <756 mmHg had relatively little effect on survival, while pressure >756 mmHg decreased survival probabilities (Figs 3A and 4A). For fish passing multiple dams the range of barometric pressures that maintained survival rates above 80% grew smaller with greater dam passages indicating a cumulative effect of barometric pressure (Figs 3B,C and 4B,C). In general, steelhead that passed multiple dams were more resilient to the effects of higher barometric pressures (>760 mmHg) than Chinook salmon (Figs 3 and 4).

Survival of Chinook and steelhead passing one dam decreased as dissolved gas concentrations increased above 113% (Figs 3D and 4D). For both species passing two dams, there is a sharp decrease in survival at 113% and then again at concentrations >120% (Figs 3E and 4E). There was a nonlinear relationship with dissolved gas for both species passing three dams, with low concentrations having a similar negative effect on survival as high concentrations (Figs 3F and 4F). Although a similar pattern exists between these species, steelhead appear more tolerant of elevated dissolved gas concentrations between 113% and 120% (Figs 3D–F and 4D–F).

Chinook passing one and two dams showed increased survival with increasing outflow discharge (Fig. 3G,H). For steelhead passing one dam, outflow discharge between 7000 and 10,000 m^3^·sec^−1^ showed the highest survival (Fig. 4G). For both species passing three dams, there is a large increase in survival at outflow discharges between 6000 and 7000 m^3^·sec^−1^ and then virtually no effect above 7000 m^3^·sec^−1^ (Figs 3I and 4I).

There was slight decrease in survival with increasing spill discharge for both species passing a single dam (Figs 3J and 4J). Fish passing three dams showed a sharp increase in survival at spill discharges ~3000 m^3^·sec^−1^ (Figs 3L and 4L). Interestingly, after averaging out the effects of other variables, survival probabilities based on spill volume alone were asymptotic around 70% and did not increase as spill discharges increased above 3000 m^3^·sec^−1^ (Figs 3J–L and 4J–L).

In general, for both species and all dam passages, survival increased as water temperatures increased up to 12**°**C; however, for temperatures above 12**°**C, survival drops quickly (Figs 3M–O and 4M–O). Steelhead survival, in general, appeared more resilient to the effects of water temperatures than Chinook salmon survival (Figs 3M–O and 4M–O).

For both species passing a single dam, fish velocities around 2 km·h^−1^ showed the highest survival (Figs 3P and 4P). For Chinook passing two dams, survival was highest around 4.5 km·h^−1^, while for steelhead, there was sharp increase in survival between 0.5 and 2 km·h^−1^ and little effect of increasing velocity past 2 km·h^−1^ (Figs 3Q and 4Q). For both species passing three dams survival increased substantially as fish velocity increased, though interestingly, survival declined slightly for Chinook salmon at velocities >3 km·h^−1^, while steelhead survival remained high for all velocities (Figs 3R and 4R).

## Discussion

The influence of biological and environmental variables on smolt survival changed depending on dam passage experience, and we observed cumulative, negative effects for fish passing multiple dams. Our analysis indicates that the ecological effects of hydropower facilities are not confined to isolated areas of deleterious environment conditions (i.e., pulse disturbance), but rather, exert a cumulative influence on migrating smolt, affecting survival throughout our study system (i.e., press disturbance). For both Chinook salmon and steelhead, atmospheric barometric pressure, dissolved gas concentrations, outflow discharge, spillway discharge, water temperature, and fish velocity were identified as most influential in terms of survival; however, the importance of these variables changed based on how many dams were experienced. For example, fish velocity had little effect on the survival of Chinook salmon passing a single dam, while survival of Chinook passing three dams was strongly negatively affected when fish travelled at low velocities.

In general, Chinook salmon and steelhead responded similarly to altered ecological conditions. We believe that the strength and influence of the altered river conditions acting on salmonids during this life stage overcomes physiological and biological differences between these species. Both species had statistically similar survival rates for each dam passage and had similar overall responses to environmental conditions. Steelhead appeared to handle the effects of dissolved gas slightly better than Chinook salmon, but this trend was not reflected in overall survival rates. These findings suggest that strategies that create more favorable ecological conditions, that improve survival for one species, will benefit other salmonid species as well. The influence of environmental variables and their implications are discussed below.

Outflow discharge has been shown to affect survival indirectly, by slowing migration and leading to increased predation and longer exposures to deleterious environmental variables (Raymond 1979; Weitkamp and Katz 1980, Giorgi et al. 1997). In our study, increasing outflow discharge did not increase survival of smolt passing one or two dams but there was a substantial benefit to smolt passing three dams (Figs 3 and 4). The effect of flow volume on smolt survival is likely more important during low flow years when delayed migration and predation risks are higher (Connor et al. 2003; Smith et al. 2003), compared to years of high flow volumes where secondary processes such as involuntary spill dominate survival patterns (this study, Raymond 1979).

Beginning in 1991, the US Army Corps of Engineers began implementing measures at Snake and Columbia River dams to increase the survival of fish populations listed under the Endangered Species Act (USACE 2011). One successful management strategy is a program of voluntary water release through spillways during juvenile outmigration periods. Smolt passage through spillways has been repeatedly shown to have the highest survival rates of any in‐river passage route (Muir et al. 2001; Budy et al. 2002). During years of high spring runoff when water flows exceed hydroelectric capacity, dams are forced into periods of involuntary spill, which result in dissolved gas concentrations that exceed the State of Oregon's water quality standard for concentrations <110% saturation (USACE 2011; Appendix S4). In our study, migrating Chinook salmon and steelhead were strongly influenced by high flow volumes and cascading effects resulting from involuntary spill through the Lower Columbia River hydrosystem.

As seen over the course of our study period, high flow volumes and involuntary spill elevate dissolved gas concentrations resulting from entrained atmospheric gasses held in solution (Johnson et al. 2005; Appendix S3). Gas concentrations >100% saturation have been shown to have both acute and chronic effects on salmonids that manifests as gas bubble trauma, which is most affected by the concentration of dissolved gas and the length of exposure (Mesa et al. 2000). While acute exposure to gas concentrations <120% are unlikely to cause direct mortality, chronic exposure, and behavioral changes to compensate for high levels of gas may indirectly increase both species' susceptibility to predation and disease (e.g., Ebel and Raymond 1976; Mesa and Warren 1997). Over the course of our study, the median dissolved gas concentrations were 116.6, 113.3, and 112.7% at the forebay of BON, TDA, and JDA, respectively, with maximum concentrations of 124.7%, 126.2%, and 131% (Appendix S3). These concentrations are well within the ranges found to cause gas bubble trauma in salmonids (Colt 1986, Mesa and Warren 1997; Mesa et al. 2000).

Multiple factors acting in concert may influence how dissolved gas concentrations will affect migrating smolt, including barometric pressure, water temperature, and fish velocity (Colt 1986, Mesa et al. 2000). The influence of atmospheric barometric pressure on dissolved gas concentrations has received little attention in recent years. The difference between atmospheric barometric pressure and total gas pressure of water is called the differential pressure (*ΔP*), where *ΔP* values <0 inhibit bubble formation and values >0 can lead to gas bubble formation in aquatic organisms (Colt 1986). Salmonids experience chronic gas bubble trauma when *ΔP* is between 38–76 mmHg and acute gas bubble trauma at levels >76 mmHg (Colt 1986). Salmonids can behaviorally adjust swimming depths to avoid *ΔP* >38 mmHg. Although *ΔP* changes throughout the season, with changing spillway discharge and barometric pressure, migration depths >2 m would compensate for all *ΔP* values calculated over our study period (Appendix S5). Swim bladder over inflation caused by sudden changes in pressure in supersaturated water may prevent smolt from reaching compensation depths (Shrimpton et al. 1988). Despite no difference in survival estimates, our partial dependence plots indicate that steelhead are slightly better adapted to an environment of elevated barometric pressures and dissolved gas concentrations compared to Chinook salmon (Figs 3 and 4).

In addition to depth compensation, migrating smolt can reduce the effect of gas bubble trauma by increasing swimming velocity, which reduces exposure times to elevated gas levels. For both species passing a single dam, all measured velocities maintained survival probabilities above 80%. For fish passing two and three dams, fish velocities <2.5 km·h^−1^ resulted in survival well below 80% (Figs 3Q–R and 4Q–R). This finding suggests that faster fish limit exposure time to sublethal levels of dissolved gas or other sources of mortality and supports our cumulative effect hypothesis.

For both species, we observed a positive relationship between survival and water temperatures between 9 and 12°C (Figs 3 and 4). At temperatures >12°C, survival decreased for both species, but this effect was stronger for Chinook salmon. Water temperatures observed between April and June were well below acute lethal levels for both species (<24°C; Sullivan et al. 2000); thus, we hypothesize that the pattern of increasing survival with increasing temperature is related to the inverse relationship between water temperature and dissolved gas concentrations. Weitkamp and Katz (1980) report that as water temperatures rise its capacity to hold dissolved gas in solution decreases, thus reducing the risk of gas bubble trauma for fish.

## Conclusions

Fish passing one, two, and three dams experience varying environmental conditions that differentially affect survival rates. The majority of Chinook salmon and steelhead smolt in the Lower Columbia River are outmigrating from upriver sites and therefore likely pass through multiple dams. While salmonids are physiologically and behaviorally adapted to wide ranges of environmental conditions, the altered state of the Lower Columbia River hydrosystem represents novel conditions for which smolts have little evolutionary context (Hicks et al. 1991). Despite being spatially isolated in the system, the temporal frequency that smolts encounter these dams equates to a press disturbance, limiting smolts' ability to recover from one set of deleterious conditions before experiencing another significant disturbance. We find tempered encouragement in the convergent responses of Chinook salmon and steelhead survival to the altered environmental conditions in the Lower Columbia River. We believe this finding indicates that management actions intended to improve smolt survival for one species will be beneficial to other salmonids.

Anthropogenic alterations within freshwater ecosystems have caused substantial impacts to aquatic organisms throughout the world. Continued monitoring and evaluation of the ecological impacts of hydroelectric development are needed to conserve current threatened and endangered species and to prevent further species loss globally.

## Conflict of Interest

None declared.

## Supporting information


**Appendix S1.** Study area and fish release and detection data.

**Appendix S2.** Acoustic transmitter life spans and survival estimates.

**Appendix S3.** Correlations among dams and between environmental variables.

**Appendix S4.** Averaged dissolved gas and outflow volumes between 2001 – 2011.

**Appendix S5.** Differential pressure and compensation depths.
