# Supplementary material for: Factors influencing the survival of outmigrating juvenile salmonids through multiple dam passages: an individual‐based approach
Source: Ecol Evol. 2016 Jul 25;6(16):5881–92. doi: 10.1002/ece3.2326 (PMC4983599; doi:10.1002/ece3.2326)
Supplement: Supplementary file 1 — Appendix S1. Study area and fish release and detection data. [file ECE3-6-5881-s001.docx]

**Appendix S1 - Study area**


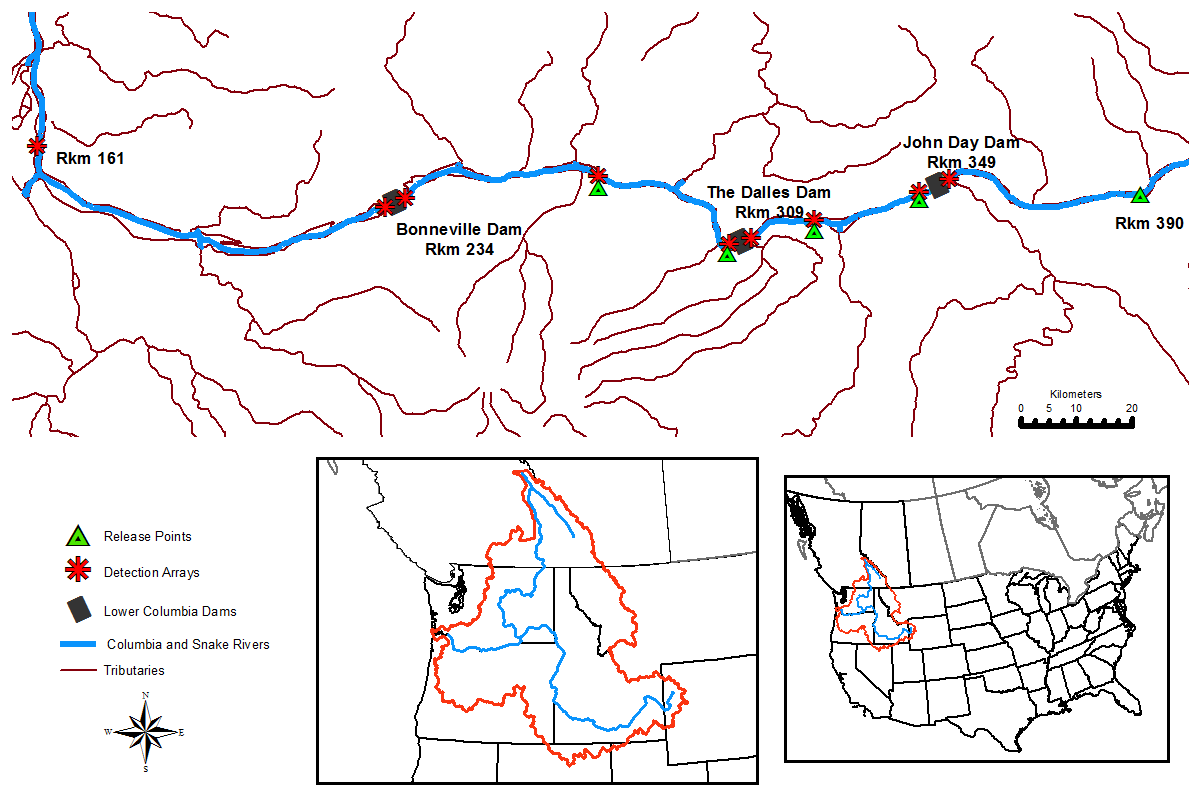


**Figure S1-1.** Map of the Lower Columbia River hydrosystem, including John Day Dam, The Dalles Dam and Bonneville Dam, autonomous detection arrays and release points for both Chinook salmon and steelhead passing one, two and three dams. Insets show the location of the Columbia River Basin within North America and major tributaries.
