# Supplementary material for: Factors influencing the survival of outmigrating juvenile salmonids through multiple dam passages: an individual‐based approach
Source: Ecol Evol. 2016 Jul 25;6(16):5881–92. doi: 10.1002/ece3.2326 (PMC4983599; doi:10.1002/ece3.2326)
Supplement: Supplementary file 2 — Appendix S2. Acoustic transmitter life spans and survival estimates. [file ECE3-6-5881-s002.docx]

**Appendix S2 - Acoustic transmitter life spans and survival estimates**

Appendix S2 shows mean acoustic transmitter life span, explanation of survival estimates and boxplot of mean survival estimates for both species (Figure S2-1).

*Acoustic Transmitter Life Spans*

Mean tag life for randomly selected acoustic transmitters was significantly different based on manufacturing lots (Skalski et al. 2012b); however the travel time that fish passed through the study area made differences between tag lots inconsequential (mean travel time for Chinook salmon and steelhead passing three dams was 3.17 days, SD + 1.73 and 3.01 days, SD + 1.87 respectively).

*Survival estimates*

Survival estimates were calculated using the Active Tag-Life-Adjusted Survival program (ATLAS v 1.5.3; Lady, Westhagen & Skalski 2010). The ATLAS program uses acoustic tag lifespan curves to estimate tag failure rates and produces adjusted and unbiased survival estimates that account for the probability of tag failure and incorporates Cormack-Jolly-Seber model assumptions (Townsend et al. 2006). Because a large fraction (99%) of fish passed through the study area in less than 20 days, all analyses other than survival estimates used data that were not adjusted by ATLAS for tag life failure and therefore differ from those reported elsewhere (Skalski et al. 2012a-c).

**
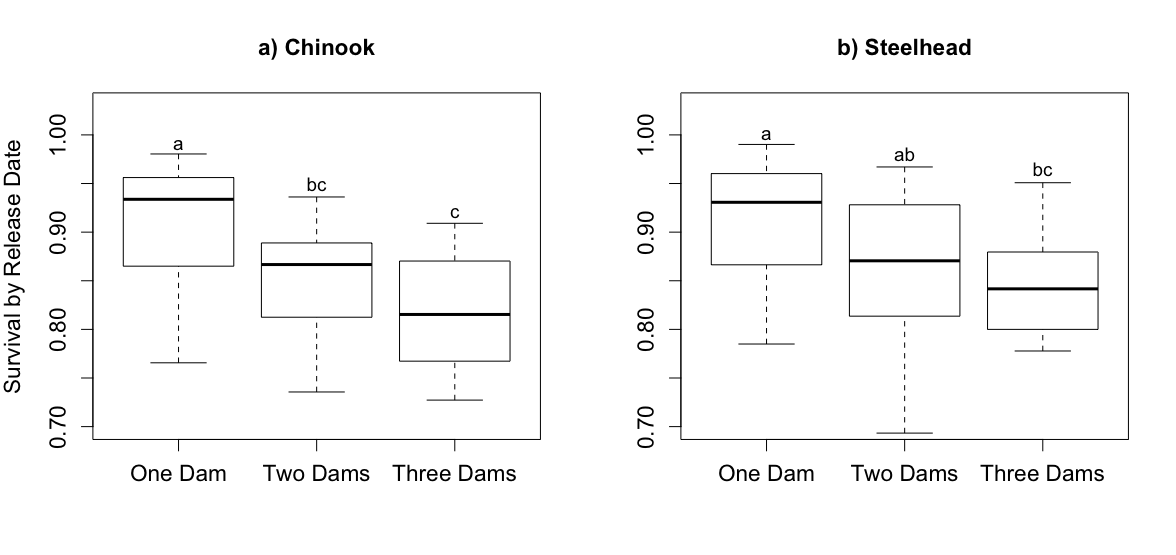
Figure S2-1.** Boxplots of the mean survival estimates of a) Chinook and b) steelhead passing one, two and three dams. Each species’ survival was assessed with a block design analysis of variance using number of dam passages as a treatment (n = 3) and blocked by released date (n = 16). Survival means with different letters are statistically significant (Tukey’s HSD, *p* < 0.05). Whiskers represent the minimum and maximum range, the box represents the inner quartiles with the median in bold.

**References**

Lady, J.M.; Westhagen, P. & Skalski, J.R. (2010) Program ATLAS 1: Active Tag Life

Adjusted Survival. <http://www.cbr.washington.edu/analysis/apps/atlas>

Townsend, R.; Skalski, J.; Dillingham, P. & Steig, T. (2006) Correcting Bias in Survival

Estimation Resulting from Tag Failure in Acoustic and Radio-telemetry Studies. *Journal of Agricultural, Biological, and Environmental Statistics*, **11**, 183–196.
