# Supplementary material for: Factors influencing the survival of outmigrating juvenile salmonids through multiple dam passages: an individual‐based approach
Source: Ecol Evol. 2016 Jul 25;6(16):5881–92. doi: 10.1002/ece3.2326 (PMC4983599; doi:10.1002/ece3.2326)
Supplement: Supplementary file 3 — Appendix S3. Correlations among dams and between environmental variables. [file ECE3-6-5881-s003.docx]

**Appendix S3 – Environmental ranges, correlations and survival estimates by dam**

Appendix S3 shows the summary statistics for measured environmental variable at each dam in the Lower Columbia River (Table S3 – 1), correlations between environmental variables measured at The Dalles Dam (Table S3 – 2; raw data) and the results from a principal components analysis (PCA) based on Chinook salmon passing one dam (Figure S3 – 1).

*Environmental variable summary statistics*

**Table S3-1 –** Minimum, maximum and mean (averaged from hourly measurements) values for each environmental variables at each dam in the Lower Columbia River hydrosystem.

|  |  | **Outflow Discharge**  **(m^3^·s^-1^)** | **Spillway Discharge (m^3^·s^-1^)** | **Temperature (°C)** | **Dissolved Gas (%)** | **Barometric Pressure (mmHg)** |
| --- | --- | --- | --- | --- | --- | --- |
| **Bonneville**  **Dam** | Min  Max  Median | 5623.7  14475.5  11248.9 | 716.4  8999.1  5132.4 | 7.1  15.8  11.8 | 103.9  124.7  116.6 | 749.0  773.0  760.0 |
| **The Dalles**  **Dam** | Min  Max  Median | 4479.7  14427.4  10972.8 | 0.0  7750.3  4202.2 | 7.1  15.8  11.9 | 104.9  126.2  113.3 | 746.0  769.0  756.0 |
| **John Day**  **Dam** | Min  Max  Median | 4989.4  15265.5  11370.6 | 852.3  7662.5  3982.7 | 6.6  15.6  11.8 | 102.9  131.0  112.7 | 742.0  766.0  753.0 |

**Table S3-2** – Results from a generalized least squares regression between the average daily means of each environmental variable recorded at The Dalles Dam compared to daily means at Bonneville Dam and John Day Dam. The high correlations indicate that environmental variables are statistically similar between dams and support our use of random forest analysis over logistic regression.

|  | **The Dalles Dam**  **X**  **Bonneville Dam** | **The Dalles Dam**  **X**  **John Day Dam** |
| --- | --- | --- |
| **Outflow**  **Discharge** | *r =* 0.96  *t* = 108.08  *p* <0.001 | *r =* 0.96  *t* = 117.63  *p* <0.001 |
| **Spillway**  **Discharge** | *r =* 0.15  *t* = 11.09  *p* <0.001 | *r =* 0.34  *t* = 15.51  *p* <0.001 |
| **Total**  **Dissolved Gas** | *r* = 0.99  *t* = 21.80  *p* <0.001 | *r =* 0.99  *t* = 20.13  *p*- <0.001 |
| **Water**  **Temperature** | *r =* 0.97  *t* = 157.59  *p* <0.001 | *r =* 0.97  *t* = 203.28  *p* <0.001 |

*Correlations between environmental variables at The Dalles Dam*

A generalized least squares (GLS) analysis for non-independent variables was conducted between the averaged daily environmental variables at The Dalles Dam in order to determine the strength of correlation among predictor variables at TDA. We examined the relationships between project outflow discharge (kcfs) and spillway discharge (kcfs), spillway discharge (kcfs) and total dissolved gas (%), and water temperature (°C) and total dissolved gas (%). Due to high degrees of autocorrelation within environmental variables (Durbin Watson Test; DW statistic = 0.27 – 0.79) time series models were run in order to determine the lag time required to account for temporal autocorrelation. The GLS models were run using the identified lag time component in order to remove autocorrelation and determine the true relationship between environmental variables.

The results of the generalized least squares analysis for non-independent variables, accounting for temporal autocorrelation, indicated that averaged daily outflow and spillway discharge were significantly correlated (*t* = 13.65; *p* < 0.001), spillway discharge and dissolved gas were significantly correlated (*t* = 3.91; *p* < 0.001) and temperature and dissolved gas were significantly correlated (*t* = 2.94; *p* = 0.004) at The Dalles Dam during our study period.

**Table S3-3 -** Eigenvalues (percent of variance explained) and eigenvectors for each principal component for Chinook passing a one dam (BON, TDA and JDA). The first four PC’s explained 95. 22% of the environmental variance and were retained following a visual inspection of the associated scree plot. An eigenvector threshold of 0.4 was used as a cut off within each principal component (bold). PC1 is most correlated with release day, outflow volume, spillway volume, water temperature and dissolved gas concentrations. PC2 and PC3 show high correlations with fish length and barometric pressure. PC4 is highly correlated with fish velocity.

|  | **PC1 = 65.02%** | **PC2 = 12.97%** | **PC3 =**  **11.95%** | **PC4 =**  **5.27%** |
| --- | --- | --- | --- | --- |
| **Release Day** | **0.43** | -0.04 | 0.03 | -0.11 |
| **Fish Length** | -0.07 | **0.70** | **0.72** | -0.15 |
| **Outflow Volume** | **0.42** | 0.01 | 0.02 | -0.15 |
| **Spillway Volume** | **0.41** | 0.15 | -0.12 | -0.28 |
| **Water Temperature** | **0.41** | -0.12 | 0.12 | 0.02 |
| **Barometric Pressure** | -0.19 | **0.61** | **-0.65** | -0.24 |
| **Dissolved Gas** | **0.40** | 0.12 | -0.13 | -0.22 |
| **Fish Velocity** | 0.32 | 0.36 | -0.12 | **0.86** |

**Figure S3-1.** Bi-plot showing the first and second principal components for Chinook salmon passing one dam in the Lower Columbia River. Eigenvectors for PC1 indicate that release day, outflow volume, spillway volume, water temperature and dissolved gas explain the 65.02% of the measured variance. PC2 and PC3 explain 12.97% and 11.95% of the variance and both are driven by fish length and barometric pressure. PC4 explains 5.27% of the variance and is explained by fish velocity. Green dots represent fish that survival, while red dots indicate fish mortality.

**Table S3-4 –** Mean, standard deviation (SD) and coefficient of variation (CV) for each

environmental variable and dam passage experience for Chinook salmon. For fish passing a single dam, the metrics were calculated from fish passing BON, JDA and TDA. For fish passing two dams, the metrics were calculated from fish passing JDA + TDA and TDA + BON. For fish passing three dams, the metrics were calculated from fish passing JDA + TDA + BON.

| **Dams** | **Variables** | **Mean** | **SD** | **CV** |
| --- | --- | --- | --- | --- |
| 1 Dam | Outflow (m^3^·s^-1^) | 10195.67 | 2886.14 | 28.30 |
| 2 Dams | Outflow (m^3^·s^-1^) | 10085.62 | 2857.91 | 28.33 |
| 3 Dams | Outflow (m^3^·s^-1^) | 10223.00 | 2813.53 | 27.52 |
| 1 Dam | Spill (m^3^·s^-1^) | 4364.00 | 1839.53 | 42.15 |
| 2 Dams | Spill (m^3^·s^-1^) | 4304.01 | 1727.80 | 40.14 |
| 3 Dams | Spill (m^3^·s^-1^) | 4331.40 | 1700.27 | 39.25 |
| 1 Dam | Temperature (°C) | 11.24 | 1.00 | 8.90 |
| 2 Dams | Temperature (°C) | 11.24 | 0.98 | 8.72 |
| 3 Dams | Temperature (°C) | 11.27 | 0.94 | 8.36 |
| 1 Dam | Dissolved Gas (%) | 113.41 | 4.50 | 3.97 |
| 2 Dams | Dissolved Gas (%) | 113.34 | 4.15 | 3.66 |
| 3 Dams | Dissolved Gas (%) | 113.50 | 3.82 | 3.36 |
| 1 Dam | Barometric Pressure (mmHg) | 757.16 | 5.43 | 0.72 |
| 2 Dams | Barometric Pressure (mmHg) | 757.30 | 4.80 | 0.63 |
| 3 Dams | Barometric Pressure (mmHg) | 756.90 | 4.11 | 0.54 |
| 1 Dam | Velocity (km·h^-1^) | 2.48 | 0.99 | 39.90 |
| 2 Dams | Velocity (km·h^-1^) | 2.48 | 0.92 | 37.44 |
| 3 Dams | Velocity (km·h^-1^) | 2.40 | 0.87 | 36.50 |
