# Supplementary material for: Factors influencing the survival of outmigrating juvenile salmonids through multiple dam passages: an individual‐based approach
Source: Ecol Evol. 2016 Jul 25;6(16):5881–92. doi: 10.1002/ece3.2326 (PMC4983599; doi:10.1002/ece3.2326)
Supplement: Supplementary file 4 — Appendix S4. Averaged dissolved gas and outflow volumes between 2001 – 2011. [file ECE3-6-5881-s004.docx]

**Appendix S4 – Dissolved Gas and Outflow Discharge Over the Past Ten Years**


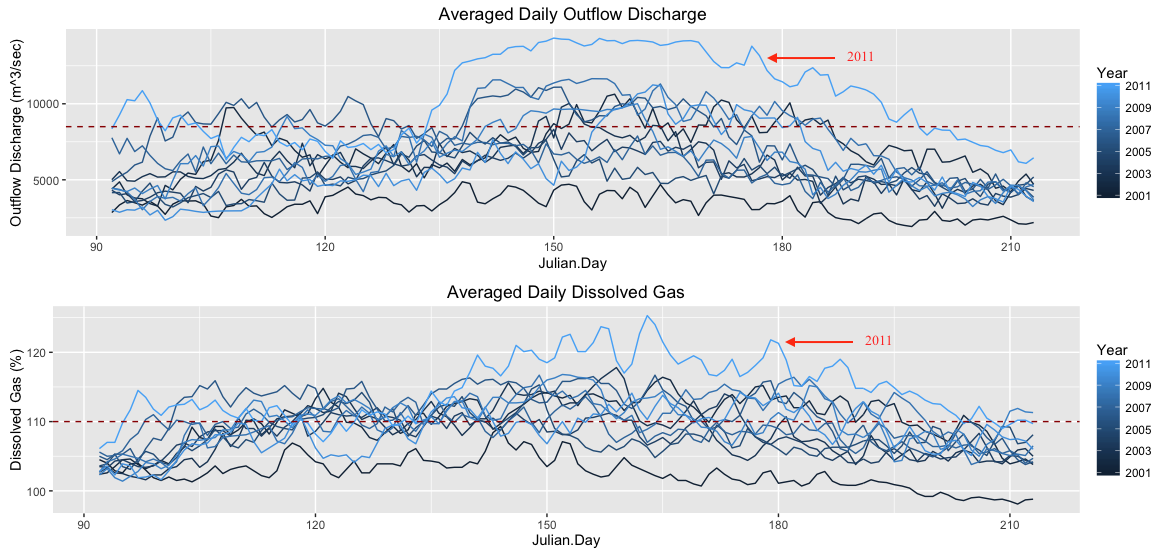


Figure S4-1. Averaged dissolved gas (%) and outflow discharge (m^3^·sec^-1^) in the Lower Columbia River Hydrosystem over the past ten years. Hourly measurements from each dam (BON, TDA and JDA) averaged from April 1 – July 31 (day of year 92 – 213). Horizontal red lines represents outflow volumes (upper panel) that would initiate involuntary spill by exceeding hydro-capacity at BON dam (>8495 m^3^·sec^-1^) and the State of Oregon’s dissolved gas concentrations water quality standard (110%).
