# Supplementary material for: Factors influencing the survival of outmigrating juvenile salmonids through multiple dam passages: an individual‐based approach
Source: Ecol Evol. 2016 Jul 25;6(16):5881–92. doi: 10.1002/ece3.2326 (PMC4983599; doi:10.1002/ece3.2326)
Supplement: Supplementary file 5 — Appendix S5. Differential pressure and compensation depths. [file ECE3-6-5881-s005.docx]

**Appendix S5 – Differential pressure (**Δ**P)**

Appendix S5 shows the daily averaged differential pressure (ΔP) that would be experienced by smolt migrating at the surface, 1m and 2m depths at each of the Lower Columbia River dams. Differential pressure is calculated as the difference between total dissolved gas pressures in river and atmospheric barometric pressure (see Colt 1983). Differential pressures >38 mmHg (shaded grey) have been shown to cause chronic gas bubble trauma and pressures >76 mmHg cause acute gas bubble trauma. Salmonid smolt can avoid harmful ΔP levels through hydrostatic compensation (swimming at deeper depths). Throughout our study period, migration depths of 2m should compensate for all elevated ΔP levels in 2011.


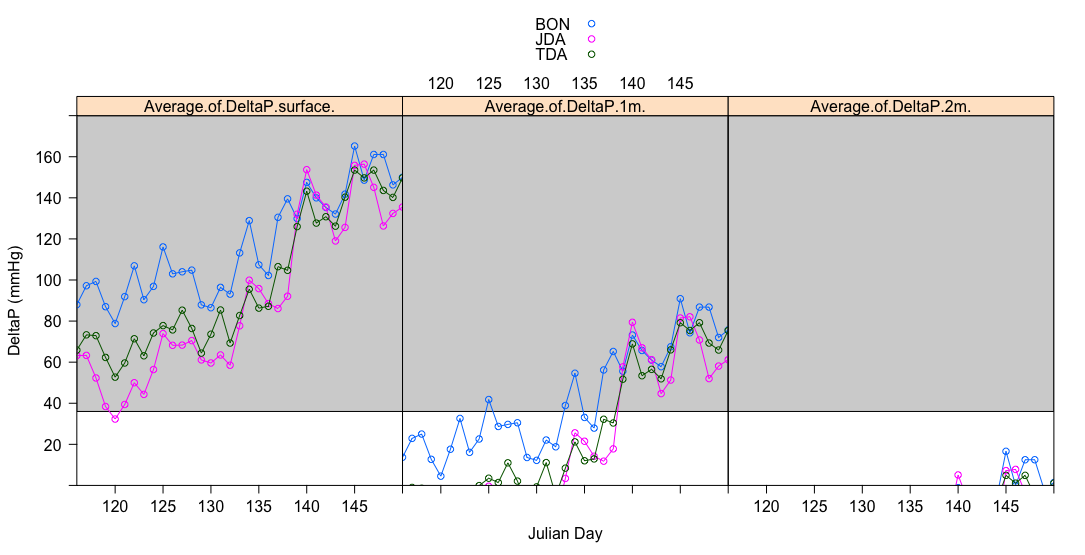


**Figure S5-1.** Averaged daily differential pressure (ΔP) experienced by smolt migrating at the (a) surface, (b) 1-m and (c) 2-m depths at each of the Lower Columbia River dams. Differential pressures >38 mmHg (shaded grey) have been shown to cause chronic gas bubble trauma and pressures >76 mmHg cause acute gas bubble trauma.
